# Supplementary material for: Targeting c-Myc with a novel Peptide Nuclear Delivery Device
Source: Sci Rep. 2020 Oct 20;10:17762. doi: 10.1038/s41598-020-73998-x (PMC7576588; doi:10.1038/s41598-020-73998-x)
Supplement: Supplementary file 1 — Supplementary Figures. [file 41598_2020_73998_MOESM1_ESM.pdf]

Targeting c-Myc with a novel Peptide Nuclear Delivery Device

Trinda Anne Ting[1+], Alexandre Chaumet[1+], and Frederic Bard[1,2*]

[1]: Institute of Molecular and Cell Biology, Singapore 138673, Singapore
[2]: Department of Biochemistry, National University of Singapore, Singapore 119077, Singapore
[+]: Participated equally in the study
[*]: Corresponding author fbard@imcb.a-star.edu.sg

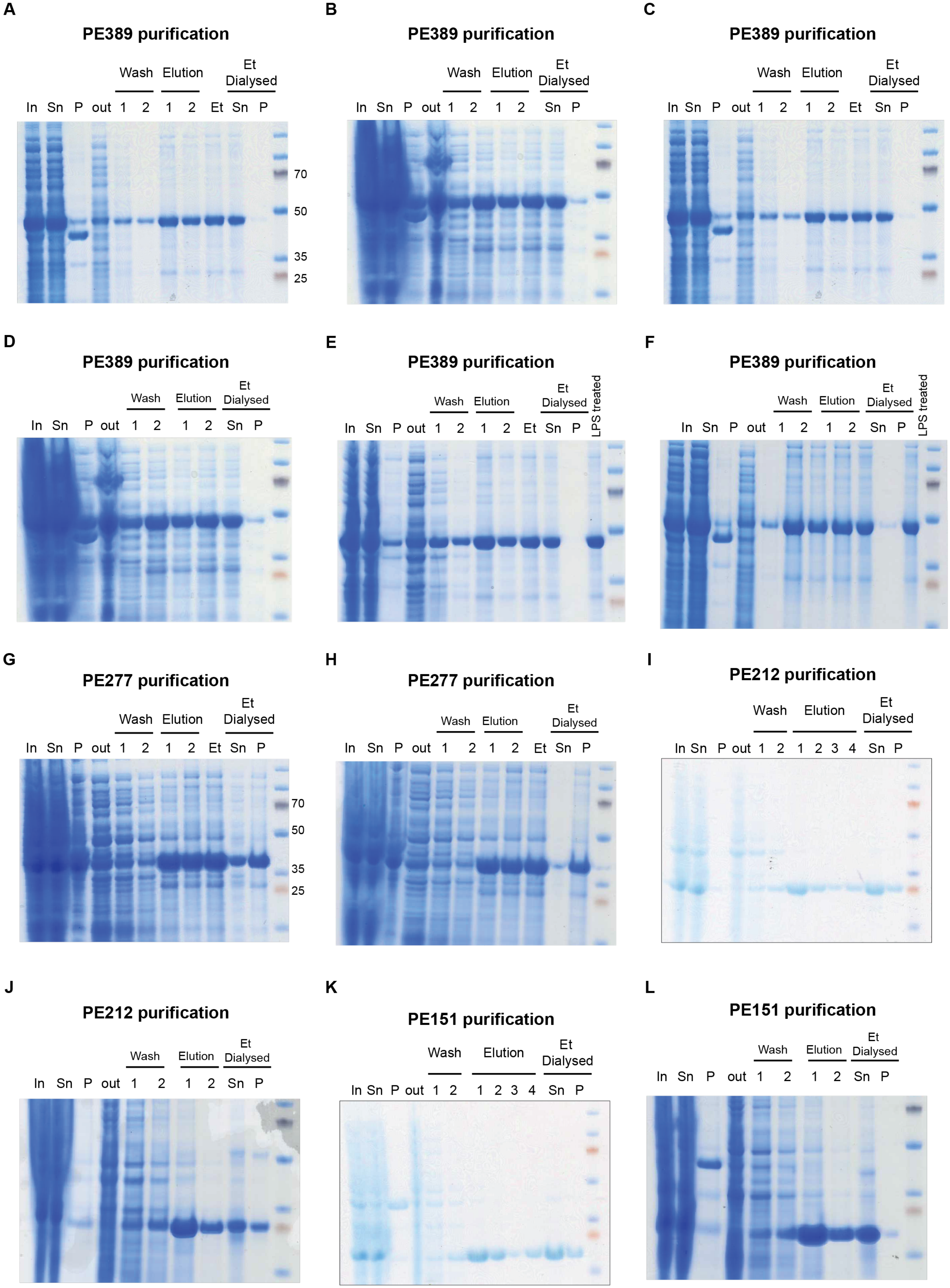

Supplementary figure 1

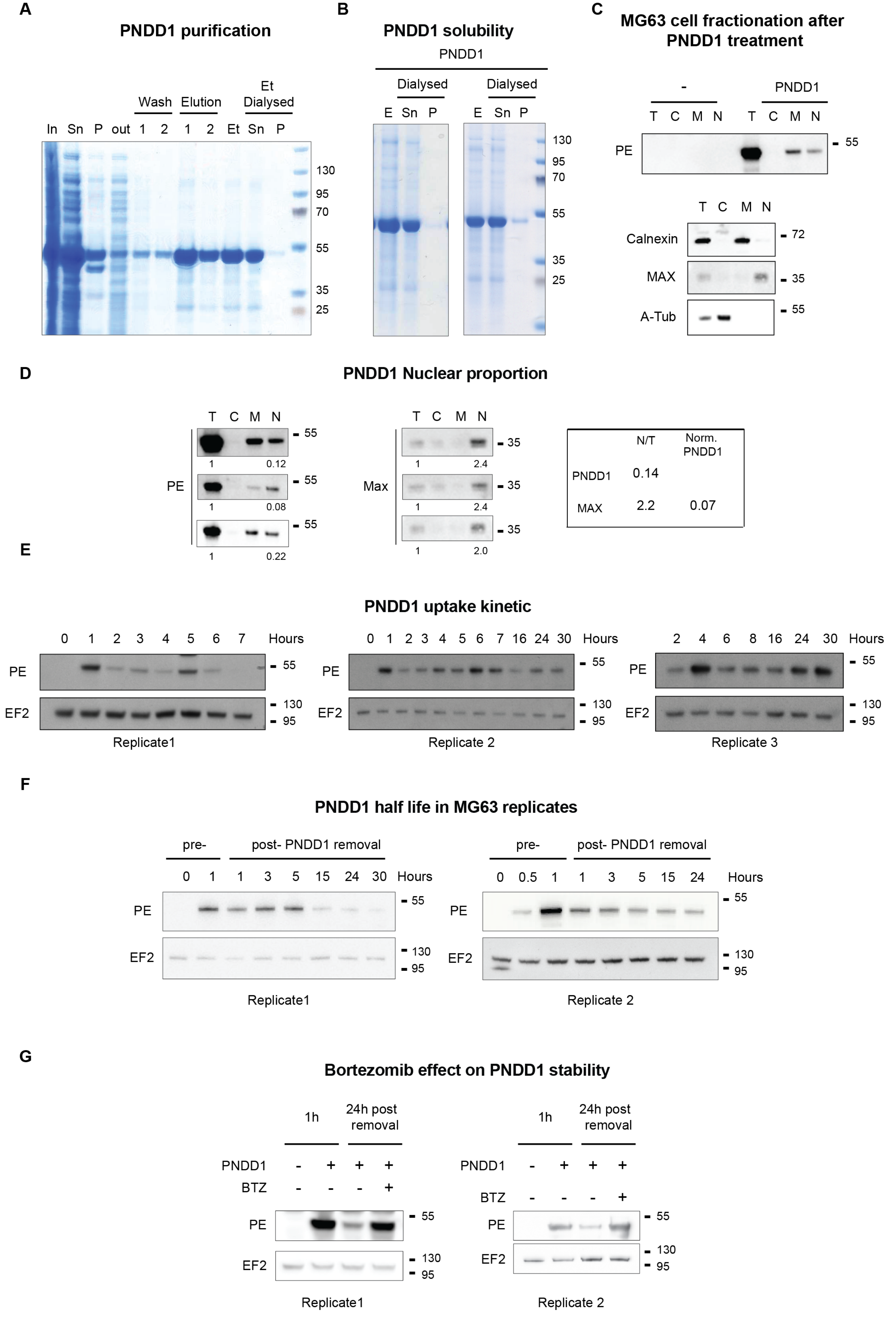

supplementary figure 2

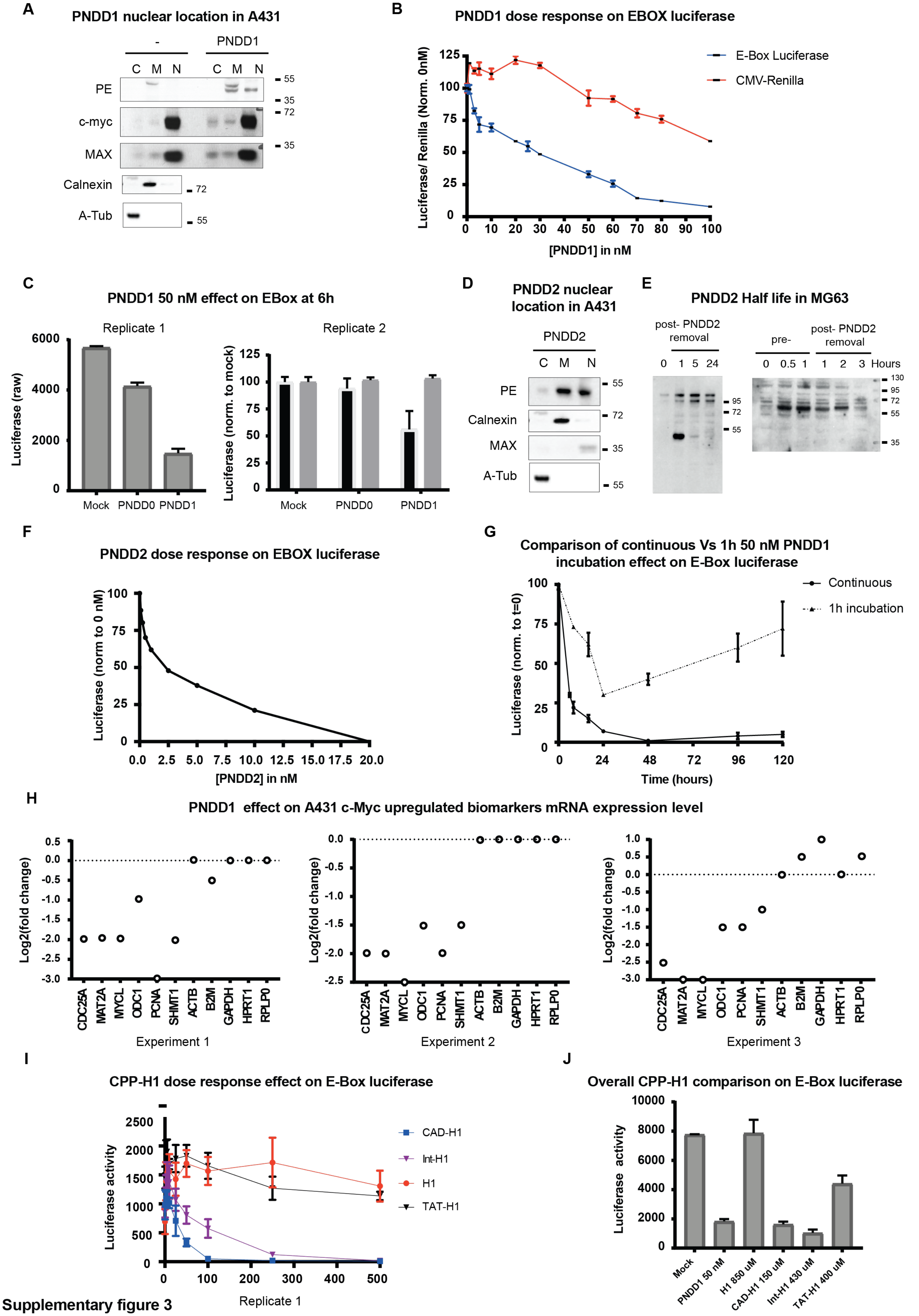

Supplementary figure 3

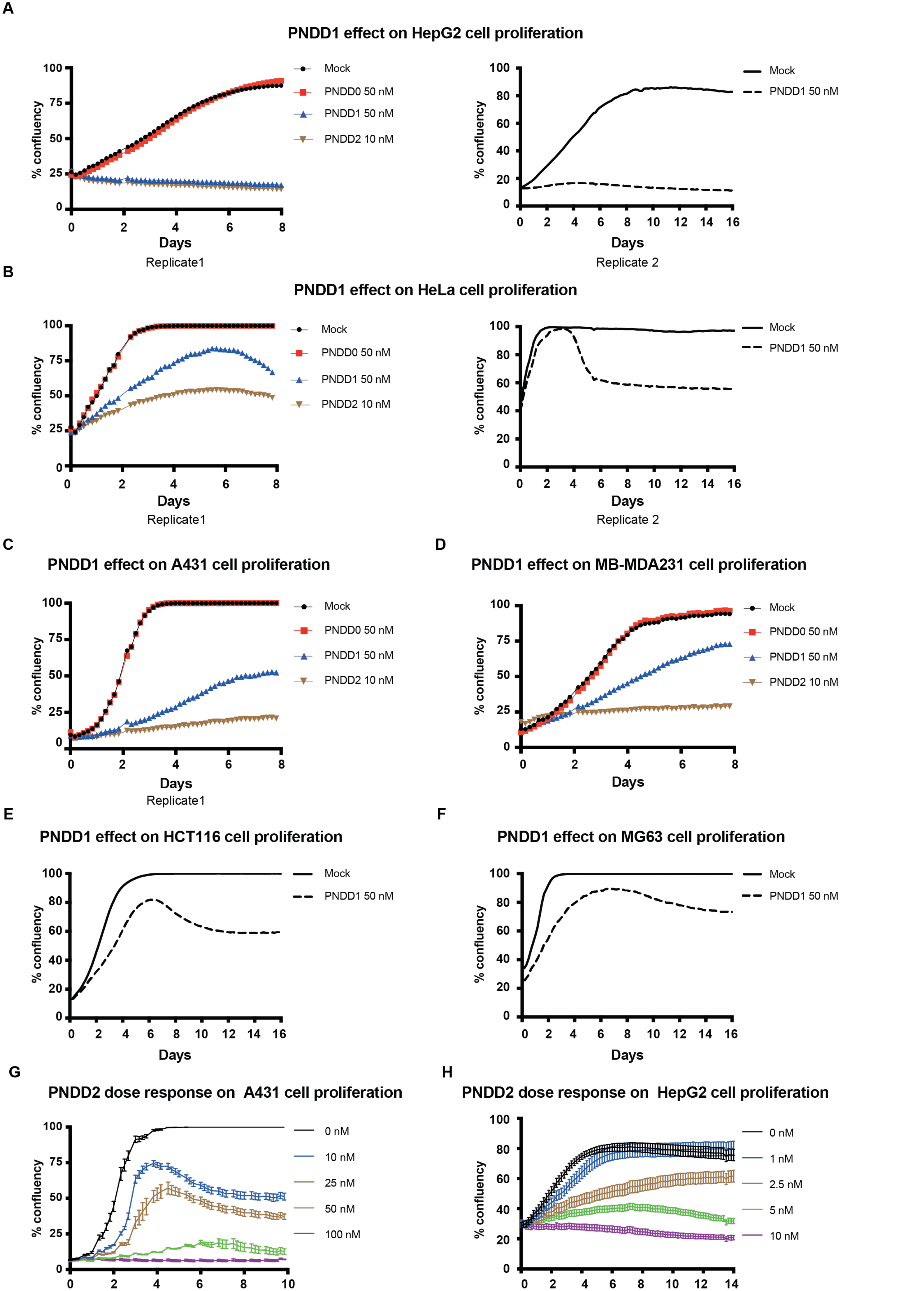

supplementary figure 4

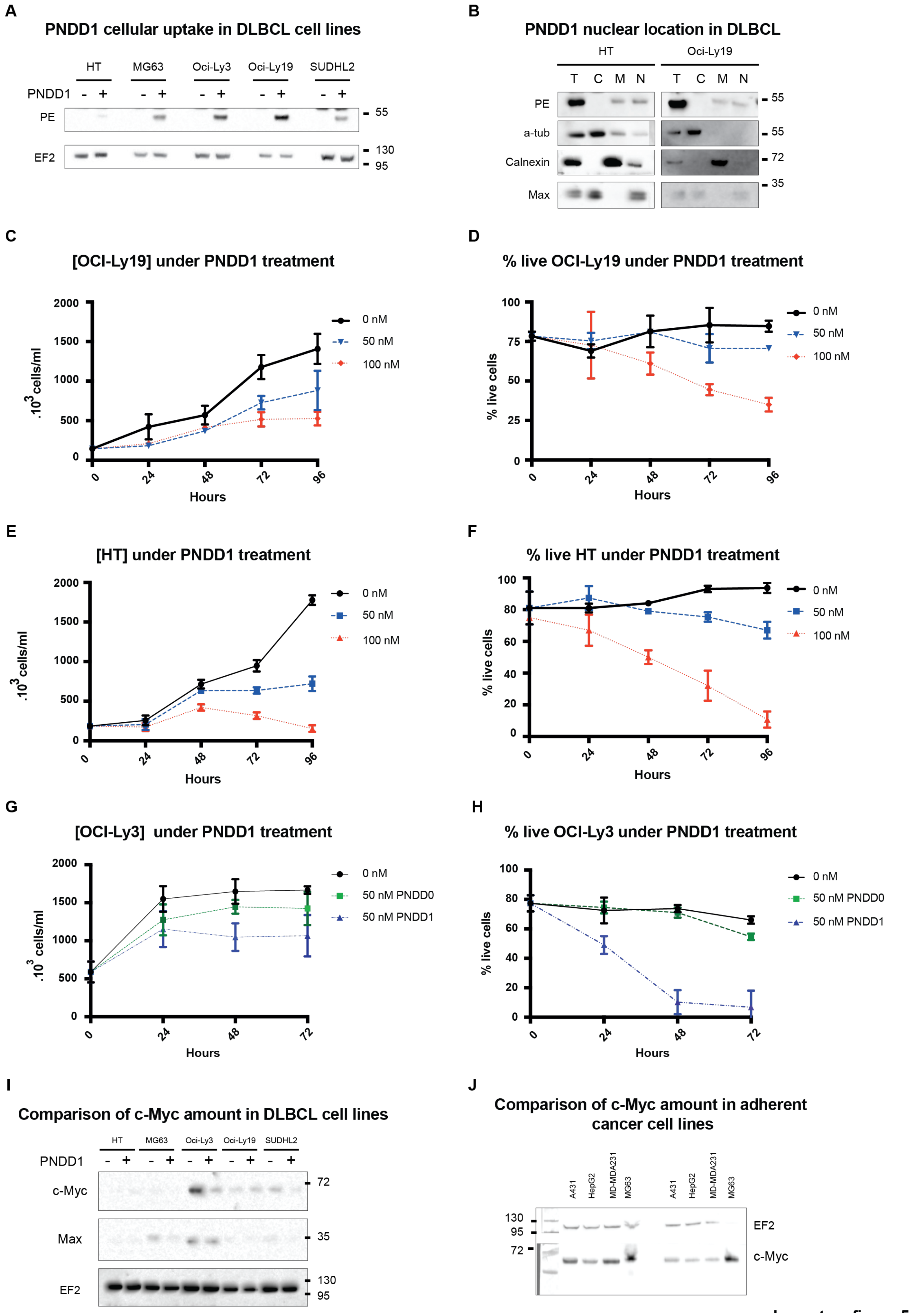

supplementary figure 5

Annex 1

A

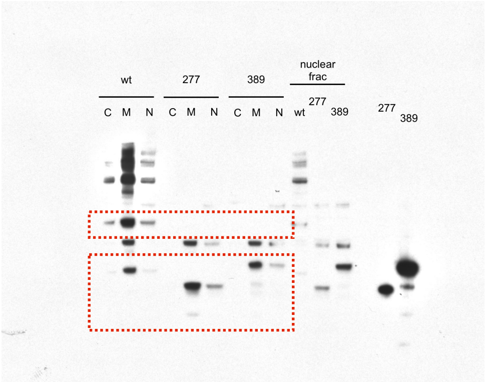

B

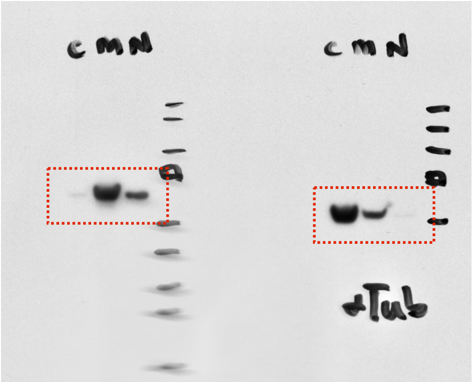

C

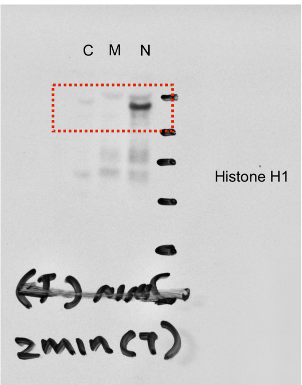

A: MG63 cell fractionation after 1 hour treatment with PE wt, PE389 or PE277 construct. C: Cytosolic fraction; M: Membrane fraction; N: Nuclear fraction.

Published area in figure1C are delimited by red dots. Figure were cropped to remove unspecific bands between the squares.

B: MG63 cell fractionation control. Left Blot: PDI for membrane fraction, right Blot: alpha tubuline for cytosolic fraction. Molecular weight are on the right. C: Cytosolic fraction; M: Membrane fraction; N: Nuclear fraction.

Published area in figure1C are delimited by red dots.

C: MG63 cell fractionation control. Histone H1 for nuclear fraction. Molecular weight are on the right. C: Cytosolic fraction; M: Membrane fraction; N: Nuclear fraction.

Published area in figure1C are delimited by red dots.

Annex 2

A

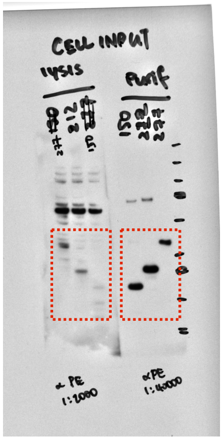

B

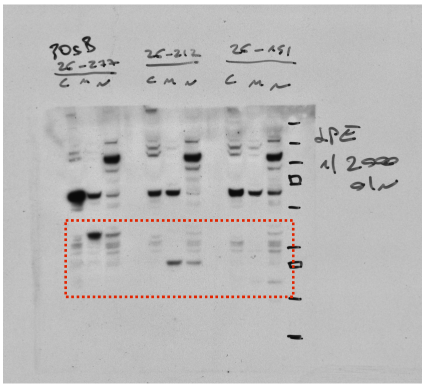

A: Left blot: MG63 were incubated with PE277, PE212, PE151 during 1 hour before cell lysis. Total extract was analyzed by western blot.

Right blot: Purified PE277, PE212, PE151 before cell incubation. Horizontal symmetry was applied.

Published area in figure1D are delimited by red dots.

B: MG63 cell fractionation after 1 hour treatment with PE151, PE212 or PE277 construct. C: Cytosolic fraction; M: Membrane fraction; N: Nuclear fraction.

Published area in figure1D are delimited by red dots. Blot was cropped to remove unspecific bands.

Annex 3

A

B

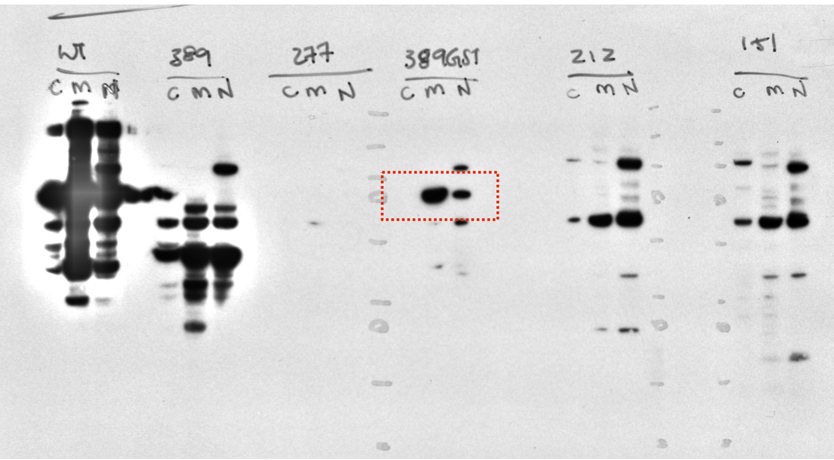

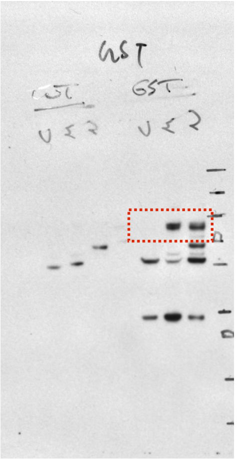

A: MG63 were incubated with PE389-GST during 1 hour before cell fractionation. Fractions were analyzed by western blot. C: Cytosolic fraction; M: Membrane fraction; N: Nuclear fraction.

Published area in figure1G are delimited by red dots. Blot was cropped to remove unspecific bands and to focus on PE389GST

B: MG63 were incubated with PE389-GST during 1 hour before cell fractionation. C: Cytosolic fraction; M: Membrane fraction; N: Nuclear fraction.

Published area in figure1G are delimited by red dots. Blot was cropped to remove unspecific bands. Blot was cropped to remove unspecific bands and to focus on PE389GST.

Annex 4

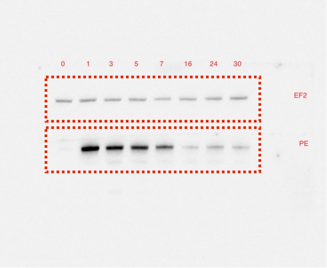

Published area in figure2 C are delimited by red dots. Blot was cropped to focus on PNDD1 and EF2. Time in hours is labeled one the top and antibodies used on the right.

Annex 5

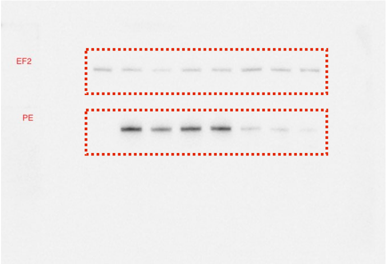

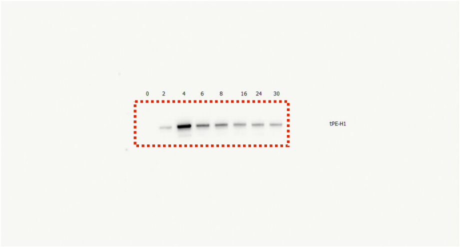

Published area in Supplementary figure 2F are delimited by red dots. Blot was cropped to focus on PNDD1 and EF2. Time in hours is labeled one the top and antibodies used on the side.

Annex 6

A

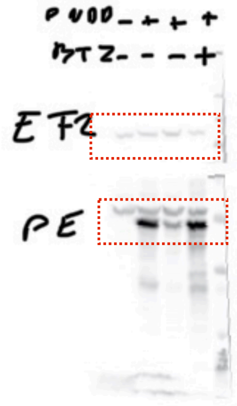

B

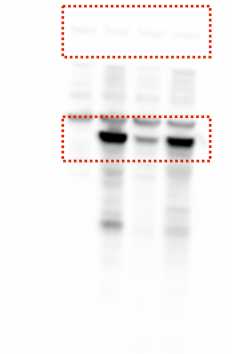

C

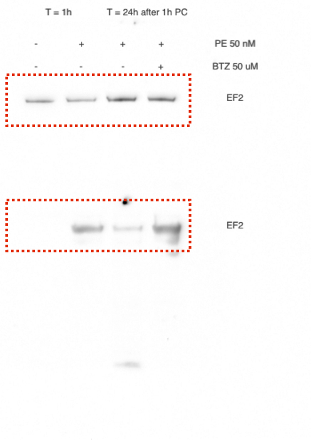

A Published area in figure 2E are delimited by red dots. Blot was cropped to focus on PNDD1 and EF2.

B and **C** Published area in Supplementary figure 2G are delimited by red dots. Replicates of figure 2E Blot was cropped to focus on PNDD1 and EF2.

Presence of treatment is indicated on the top of the blot. Top blot: EF2, lower Blot: PNDD1.

Annex 6

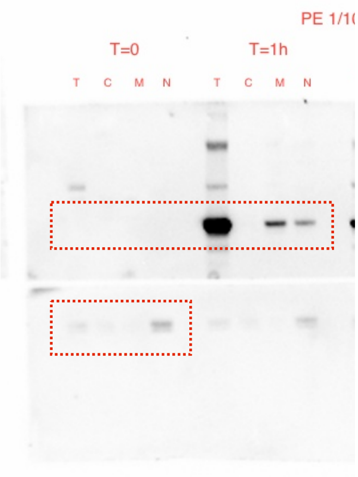

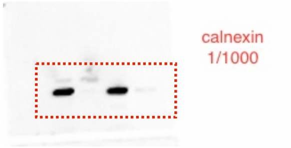

Published area in supplementary figure 2C are delimited by red dots. Blot was cropped to focus on PNDD1 and the fractionation controls. T: total; C: Cytosolic fraction; M: Membrane fraction; N: Nuclear fraction.

Annex 7

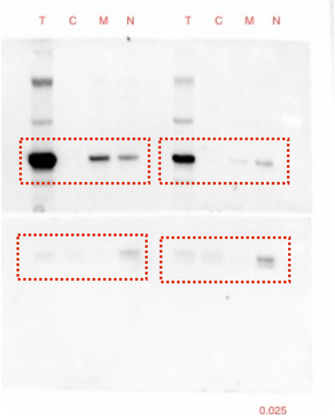

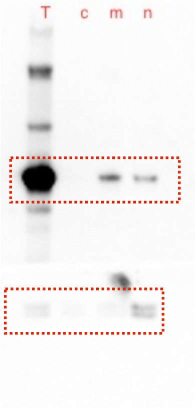

Published area in supplementary figure 2D are delimited by red dots. Blot was cropped to focus on PNDD1 and Max. T: total; C: Cytosolic fraction; M: Membrane fraction; N: Nuclear fraction.

Annex 8

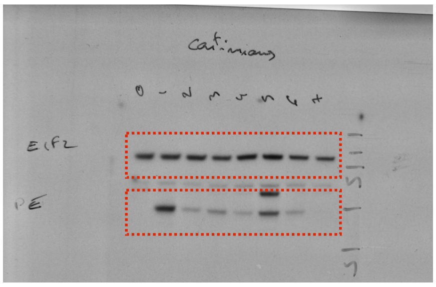

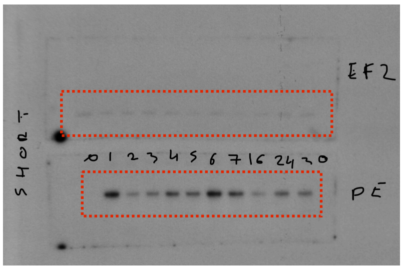

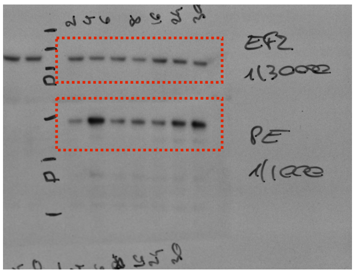

Published area in supplementary figure 2E are delimited by red dots. Blot was cropped to focus on PNDD1 and EF2.

Annex 9

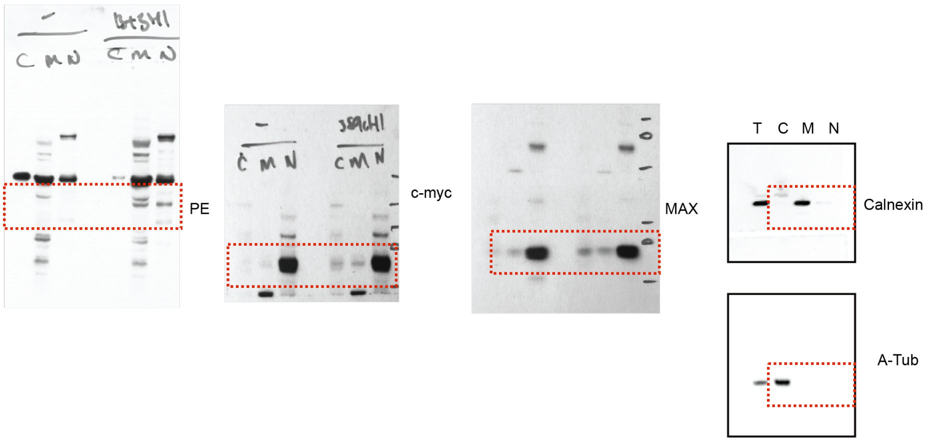

Published area in supplementary figure 3A are delimited by red dots. Blot was cropped to focus on PNDD1 and fractionation controls. T: total; C: Cytosolic fraction; M: Membrane fraction; N: Nuclear fraction.

Annex 10

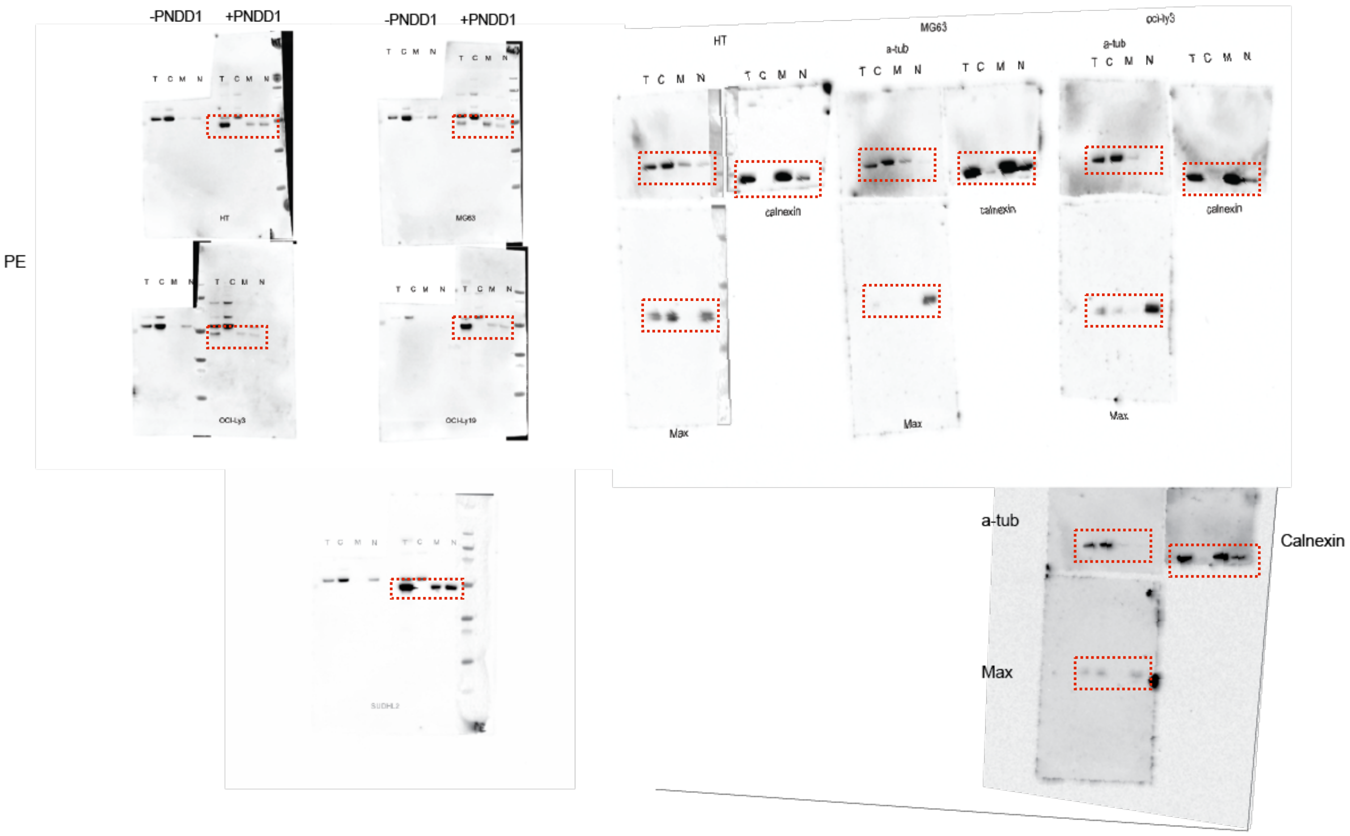

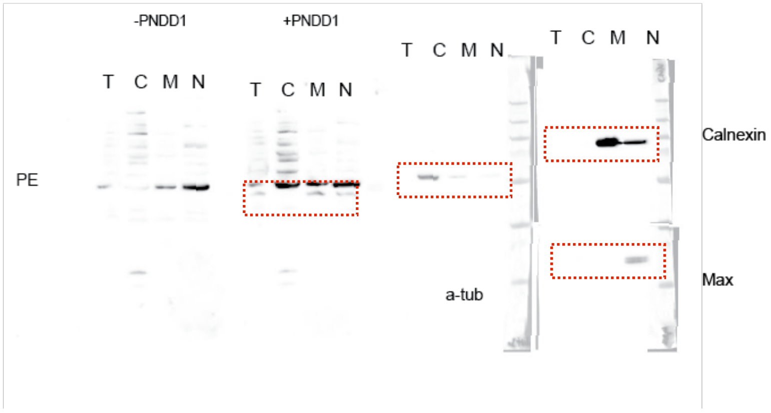

Published area in Figure 5A and supplementary figure 5B are delimited by red dots. Blot was cropped to focus on PNDD1 and fractionation controls. T: total; C: Cytosolic fraction; M: Membrane fraction; N: Nuclear fraction.

Annex 11

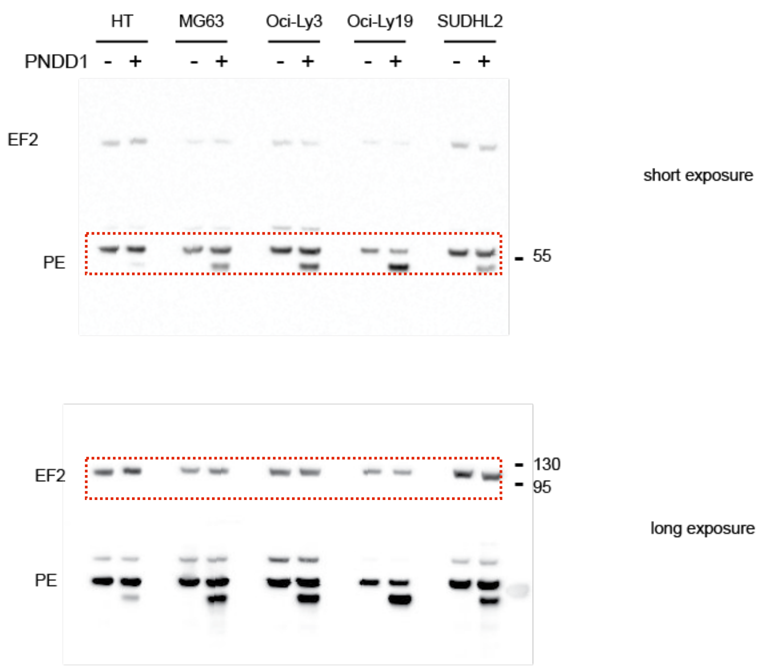

Published area in supplementary figure 5A are delimited by red dots. Blot was cropped to focus on PNDD1 and EIF2
