# Supplementary material for: Targeting c-Myc with a novel Peptide Nuclear Delivery Device
Source: Sci Rep. 2020 Oct 20;10:17762. doi: 10.1038/s41598-020-73998-x (PMC7576588; doi:10.1038/s41598-020-73998-x)
Supplement: Supplementary file 2 — Supplementary Legends. [file 41598_2020_73998_MOESM2_ESM.docx]

**Supplementary figure legends**

**Figure S1: PNDD purification.**

**A-F** PE389 purification; **G-H** PE277 purification; **I-J** PE212 purification; **K-L** PE151 purification. In: Input, total bacterial extract; Out: Output, unbound proteins; Sn: Supernatant; P: Pellet; Et: Elution total, E1+E2.

**Figure S2: PNDD1 characterisation.**

PNDD1 solubility: (**A**) PNDD1 purification followed by SDS-PAGE stained with coomassie blue. T: Total, Sn: supernatant, P: Pellet, Out: output, Et: Elution total. Molecular weights are shown on the right. Western blot was cropped horizontally to point specific bands, cropped portions are delimited with black squares, full blot is presented in Annexe 6 (**B**) Coomassie staining replicates showing PNDD1 solubility after dialysis and spin down. E: Elute before dialysis and centrifugation; Sn: Supernatant after centrifugation; P: Pellet after centrifugation. Molecular weights are shown on the right (left panel). PNDD1 subcellular distribution: (**C**) MG63 cell fractionation after 1 hour treatment with PNDD1. Alpha-tubulin (A-Tub), calnexin and MAX are used as fraction controls for respectively C: Cytosolic fraction; M: Membrane fraction; N: Nuclear fraction. Representative western blot of 3 independent experiments. (**D**) Left panel: PNDD1 cell fractionation. Quantification of ratio nucleus/total extract (under corresponding band). PNDD1 N/T average ratio = 0.14. C: Cytosolic fraction; M: Membrane fraction; N: Nuclear fraction. Antibodies are labelled on the left of each blot. Molecular weight is shown on the right. Middle panel: Max cell fractionation. Quantification of ratio nucleus/total extract (under corresponding band). Max N/T average ratio = 2.2 C: Cytosolic fraction; M: Membrane fraction; N: Nuclear fraction. Antibodies are labelled on the left of each blot. Molecular weight is shown on the right. Western blot was cropped horizontally to point specific bands, cropped portions are delimited with black squares, full blot is presented in Annexe 7. Normalisation PE nuclear proportion over Max: 0.14/2.2= 6.3%. Right panel: table summarizing PNDD1 N/T ration, Max N/T ration and PNDD1 normalisation over Max. PNDD1 stability: (**E**) PNDD1 uptake kinetic during continuous incubation. Total cell lysates were lysed at different time points up to 30 hours and PNDD1 remaining amount was analysed by western blot to determine its half life in cells. EF2 was used as a loading control. Molecular weights are shown on the right. Western blot was cropped horizontally to point specific bands, cropped portions are delimited with black squares, full blot is presented in Annexe 8 (**F**) Replicates 1 and 2 of Fig. 2B. PNDD1 was incubated 1 hour (pre-) with MG63 cells before washing with PBS and replacing with media (post-). Total cell lysates were lysed at different time points up to 30 hours and PNDD1 remaining amount was analysed by western blot to determine its half life in cells. EF2 was used as a loading control. Molecular weights are shown on the right. Western blot was cropped horizontally to point specific bands, cropped portions are delimited with black squares, full blot is presented in Annexe 5. Representative western blot of 3 independent experiments. (**G**) Replicates of Fig. 2E. MG63 cells were incubated with PNDD1 during 1 hour with or without Bortezomib before wash of PE. As a control, MG63 cells were incubated continuously during 24 hours with PNDD1. EF2 was used as a loading control. Molecular weights are shown on the right. Triplicate experiments are shown.

**Figure S3: PNDD1 and PNDD2 effect on c-myc controlled gene expression**

(**A**) A431 cell fractionation after 1 hour PNDD1 treatment. A-Tub, calnexin and Max are used as fraction controls for respectively C: Cytosolic fraction; M: Membrane fraction; N: Nuclear fraction. Antibodies are labelled on the left of each blot. Molecular weight is shown on the right. Western blot was cropped horizontally to point specific bands, cropped portions are delimited with black squares, full blot is presented in Annexe 9. Representative western blot of 3 independent experiments. (**B**) PNDD1 dose response effect on E-Box Firefly luciferase and CMV Renilla luciferase after 6 hours incubation. (**C**) Replicates of A431 cells expressing Firefly luciferase (black bars) and Renilla (grey bars) under CMV promoter control treated for 6 hours with 50 nM PNDD0. Error bars are s.d. (**D**) A431 cell fractionation after 1 hour PNDD2 treatment. A-Tub, calnexin and Max are used as fraction controls for respectively C: Cytosolic fraction; M: Membrane fraction; N: Nuclear fraction. Antibodies are labelled on the left of each blot. Molecular weight is shown on the right. Representative western blot of 3 independent experiments. (**E**) PNDD2 dose response effect on E-Box Firefly luciferase activity after 6 hours continuous incubation. Representative of 3 independent experiments. (**F**) Firefly luciferase activity at different time points after continuous incubation (solid line) or after 1 hour treatment followed by a wash (dotted line). Error bars at s.d. (**G**) Replicates of A431 cells treated with PNDD1 overnight before RNA extraction. mRNA transcript levels of genes regulated by c‑Myc were quantified by RT-PCRQ and compared with or without PNDD1 treatment. Housekeeping mRNAs (HPRT1, GAPDH) whose expression are not regulated by c-Myc are analysed in the same manner. Y axis shows the mRNA log2(fold change). Upregulated genes appear with negative log2 (fold change) compared to housekeeping genes. Results are significant below -1.5. Error bars at s.d. (**H**) Replicate of CPP-H1 effect on Firefly luciferase activity. Error bars at s.d. (**I**) Comparative optimum to obtain 80% Firefly luciferase activity decrease after 6 hours treatment with cell targeting peptides (CPP) fused to H1 and PNDD1 on A431 cells expressing Firefly luciferase under E-Box promoter control. X axis is shown in Log Cadherin (CAD; LLIILLRRRIRKQAHAHSK;) EC_50_=75 mM, Antenapedia (Int; RQIKIWFQNRRMKWKK) EC_50_=200 mM and TAT (GRKKRRQRRRPPQ) EC_50_=500 mM. Error bars at s.d.

**Figure S4**

Cell lines sensitivity to PNDD1. Comparison of PNDD1 50 nM effect on cell proliferation of (**A**) HepG2, (**B**) HeLa, (**C**) A431, (**D**) MB-MDA231 cells. Effect of PNDD1 50 nM on cell proliferation of (**E**) HCT116 and (**F**) MG63.

**Figure S5**

(**A**) PNDD1 cellular uptake in HT, Oci-Ly3, Oci-Ly19, SUDHL2 and MG63 (control) cells. EF2 was used as a loading control. Western blot was cropped horizontally to point specific bands, cropped portions are delimited with black squares, full blot is presented in Annexe 10. Molecular weights are shown on the right. Antibodies are labelled on the left of each blot. (**B**) PNDD1 subcellular cell fractionation of HT and Oci-Ly19 cells. Oci-Ly19 cells were incubated with 50 or 100 nM PNDD1 during 4 days. Western blot was cropped horizontally to point specific bands, cropped portions are delimited with black squares, full blot is presented in Annexe 11. (**C, E, G**) Cell density was recorded every 24 hours. (**D, F, H**) percentage of live cells was recorded every 24 hours. Error bars are s.d. Representative of 3 independent experiments. (**I**) DLBCL cell lines were analysed to compare c-Myc and Max amount. MG63 cells were used as a reference to previous experiments. EF2 was used as a loading control. Antibodies are labelled on the left of each blot. Molecular weight is shown on the right. (**J**) A431, HepG2, MB-MDA231 and MG63 cell lines were analysed to compare c-Myc and Max amount. EF2 was used as a loading control. Antibodies are labelled on the right of each blot. Molecular weight is shown on the left. Duplicate experiments were loaded on the same gel.
